# Effect of compliance to continuous positive airway pressure on exacerbations, lung function and symptoms in patients with chronic obstructive pulmonary disease and obstructive sleep apnea (overlap syndrome)

**DOI:** 10.1111/crj.13580

**Published:** 2023-01-12

**Authors:** Athanasios Voulgaris, Kostas Archontogeorgis, Stavros Anevlavis, Michail Fanaridis, Marios E. Froudarakis, Sofia Schiza, Paschalis Steiropoulos

**Affiliations:** ^1^ Department of Pneumonology, Medical School Democritus University of Thrace Alexandroupolis Greece; ^2^ MSc Program in Sleep Medicine, Medical School Democritus University of Thrace Alexandroupolis Greece; ^3^ Sleep Disorders Unit, Department of Respiratory Medicine, Medical School University of Crete Heraklion Greece

**Keywords:** chronic obstructive pulmonary disease, compliance, continuous positive airway pressure, exacerbations, obstructive sleep apnea, overlap syndrome

## Abstract

**Introduction:**

Patients with overlap syndrome (OS), that is obstructive sleep apnea (OSA) and chronic obstructive pulmonary disease (COPD), are at increased risk of acute exacerbations related to COPD (AECOPD). We assessed the effect of CPAP compliance on AECOPD, symptoms and pulmonary function in OS patients.

**Methods:**

Consecutive OS patients underwent assessment at baseline and at 12 months under treatment with CPAP of: AECOPD and hospitalizations, COPD Assessment Test (CAT) and modified British Medical Research Council (mMRC) questionnaires, pulmonary function testing and 6‐min walking test (6MWT).

**Results:**

In total, 59 patients (54 males) with OS were followed for 12 months and divided post hoc according to CPAP compliance into: group A with good (≥4 h CPAP use/night, *n* = 29) and group B with poor (<4 h CPAP use/night, *n* = 30) CPAP compliance. At 12 months, group A showed improvements in FEV_1_ (*p* = 0.024), total lung capacity (*p* = 0.024), RV/TLC (*p* = 0.003), 6MWT (*p* < 0.001) and CAT (*p* < 0.001). COPD exacerbations decreased in patients with good CPAP compliance from baseline to 12 months (17 before vs. 5 after, *p* = 0.001), but not in those with poor compliance (15 before vs. 15 after, *p* = 1). At multivariate regression analysis, COPD exacerbations were associated with poor CPAP compliance (β = 0.362, 95% CI: 0.075–0.649, *p* = 0.015).

**Conclusions:**

When compared to poorly compliant patients, OS patients with good CPAP compliance had a lower number of AECOPD and showed improved lung function and COPD related symptoms.

## Graphical abstract

This study assessed the effects of continuous positive airway pressure (CPAP) compliance on acute exacerbations of COPD, symptoms and pulmonary function in patients with overlap syndrome (OS). In total, 59 patients (54 males) with OS were followed for 12 months and divided post hoc according to CPAP compliance into those with good (≥4 h CPAP use/night, *n* = 29) and those with poor (<4 h CPAP use/night, *n* = 30) CPAP compliance. COPD exacerbations decreased in patients with good CPAP compliance from baseline to 12 months (17 before vs. 5 after, *p* = 0.001), but not in those with poor compliance (15 before vs. 15 after, *p* = 1).

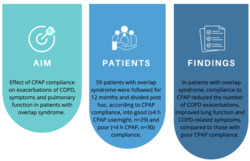

## 1 INTRODUCTION

Obstructive sleep apnea (OSA) and chronic obstructive pulmonary disease (COPD) are highly prevalent in the general population, with an occurrence of approximately 10% for each.^1^ Co‐occurrence of OSA and COPD, that is overlap syndrome (OS), was first introduced by Flenley in 1985.^2^ The prevalence of OS is estimated at 1–3.6% in the general population, while a higher occurrence of OS is evident among patients diagnosed with either OSA or COPD.^3^ Patients with OS report poorer quality of life than age‐matched COPD patients and are more likely to suffer from hypoxemia during sleep compared with OSA patients.^3^ Furthermore, OS patients have a higher risk of death and COPD exacerbations when OSA is left untreated, as compared with COPD individuals,^4^ while increased healthcare utilization is more often required in OS than COPD patients.^5^

Patients with COPD can have a rather stable course of disease; however, exacerbations may occur, which are characterized as an acute worsening of respiratory symptoms that necessitates a change in COPD current treatment or hospital admission.^6^ Exacerbations represent a significant medical and public healthcare burden, as they are related to poor quality of life, disease progression and worse survival.^7^ The Global Initiative for Chronic Obstructive Lung Disease (GOLD) 2017 report updated the ABCD assessment tool to stage patients with COPD into the ABCD categories, according to respiratory symptoms and history of exacerbations/hospitalizations in the past year.^8^

Treatment of COPD exacerbations aims at reducing their negative impact on afflicted patients and at minimizing the risk for development of future events.^7^ Application of positive airway pressure (PAP), mainly the bilevel mode, has shown benefit in several outcomes of COPD exacerbations, including mortality and need for intubation,^8^ and it is also a valid option in the management of stable COPD, particularly when daytime hypercapnia is present.^9^

In patients with OS, continuous positive airway pressure (CPAP) effectively treated OSA, improved pulmonary function and reduced mortality and risk of COPD exacerbations.^4^, ^10^ Until now, the effects of CPAP treatment on acute exacerbations of COPD and symptoms in—untreated for OSA—OS patients have not been fully elucidated.^11^ More specifically, it is under investigation whether good compliance to CPAP could confer an additive protective role on COPD outcomes, as it has been demonstrated with other OSA‐related complications.^12^

Therefore, aim of the present study was to investigate the effect of compliance to CPAP treatment, in COPD patients with newly diagnosed OSA, on the number of COPD exacerbations and hospitalizations, and on patients' symptoms related to COPD, pulmonary function and exercise capacity following one‐year treatment with CPAP.

## 2 MATERIALS AND METHODS

### 2.1 Study design

This prospective observational study was performed in a tertiary university hospital (University General Hospital of Alexandroupolis, Greece) from November 2017 to June 2021. Prior to study initiation, the protocol was approved by the institutional ethics committee (IRB:14‐1‐27.01.2017), and all procedures were conducted in accordance with the Helsinki Declaration of Human Rights.^13^ Written informed consent was obtained from all participants upon enrollment to the study. The protocol was registered in the German Clinical Trials Register (ID: DRKS00012709).

### 2.2 Participants

Consecutive COPD patients, referred to the Sleep Unit of our Department for evaluation of sleep disordered breathing from November 2017 to June 2020 and diagnosed with OSA (Αpnea hypopnea index, AHI ≥ 5/h), were prospectively enrolled in the study. Eligible patients were aged 40 years or older; were current or ex‐smokers with at least 10 pack‐year smoking history; had a diagnosis of stable COPD in the last month and received optimal bronchodilation treatment before the sleep study as per guidelines^8^; were able to understand and complete the study related procedures.

The following exclusion criteria were applied: central sleep apnea syndrome; recent infection and/or exacerbation history in the previous 3 months; respiratory acidosis or severe hypercapnia in wakefulness (pCO_2_ > 55 mmHg) in need for admission and ventilatory support with Bilevel Positive Airway Pressure; coexistent asthma; oral corticosteroid and immunomodulatory medications; active cancer; unstable cardiovascular disease (CVD) and any unstable concurrent disease; and/or clinically significant laboratory abnormalities.

### 2.3 Baseline measurements

#### 2.3.1 Clinical assessment

A detailed medical history regarding the presence of chronic respiratory symptoms, sleep habits, comorbidities, current medication, and smoking status was obtained. In addition, anthropometric characteristics, namely, height, weight, body mass index, and neck, waist and hip circumference, were recorded at initial evaluation.

Daytime sleepiness was assessed with the Greek version of the Epworth Sleepiness Scale (ESS).^14^ ESS evaluates the probability of falling asleep in a variety of daily circumstances; the maximum total score is 24, and scores above 10 are considered indicative of excessive daytime sleepiness.

#### 2.3.2 Pulmonary function

Pulmonary function was assessed by pulmonary function testing (PFTs) (MasterScreen Body, JAEGER®, Germany) according to ERS/ATS specifications and by analysis of arterial blood gases (ABGs) collected from the patients' radial artery, (ABL3000 auto‐analyser, ≥Radiometer Co., Tokyo, Japan). All PFTs were performed in accordance with the ATS/ERS standardized criteria for spirometry.^15^ Exercise capacity was also recorded by the 6‐min walking test (6MWT) according to ERS/ATS guidelines.^16^

#### 2.3.3 Blood samples

Venous blood samples were collected the morning after PSG following at least 8 h of fasting. Biochemical parameters of renal function and lipid profile, as well as glucose and C‐reactive protein (CRP) serum levels were measured using an automated analyser. Serum biochemical parameters were determined using a commercial radioimmunoassay kit and the manufacturer's specifications the day of blood sampling (DiaSorin, Stillwater, MN, USA).

#### 2.3.4 Diagnosis of COPD and exacerbations

COPD diagnosis was set at the presence of a post‐bronchodilator FEV_1_/FVC ratio of less than 0.7 in PFT, combined with chronic symptoms, such as dyspnea, cough and/or sputum production, and in association with a history of exposure to risk factors for COPD.^8^ When the diagnosis of COPD was established, participants were categorized into the “ABCD” groups according to the GOLD 2017 classification.^8^ ABCD classification comprises the combined COPD assessment criteria and briefly includes the following: (a) the presence of symptoms with the COPD Assessment Test (CAT) and the Modified British Medical Research Council (mMRC) questionnaires and (b) the history of exacerbations and hospitalizations in the last year. CAT consists of 8 items, each of which receives a score from 0 (best) to 5 (worse), and the sum of scale values for all 8 items gives a total score ranging from 0 to 40.^17^ Grade of dyspnea was assessed by mMRC dyspnea scale, which is a scale rating dyspnea from 0 (no dyspnea) to 4 (too dyspneic to do any exercise).^18^ Acute exacerbations of COPD (AECOPD) were defined moderate when antibiotics and/or oral corticosteroids were introduced to standard therapy and severe when they led to visit the emergency department or to hospital admission. In the present study, only moderate and severe exacerbations were recorded based on patient's medical records at initial evaluation and after 12 months of treatment with CPAP.

#### 2.3.5 Polysomnography

An attended overnight polysomnography (PSG) from 22:00 to 06:00 (Alice® 4, Philips Respironics, Murrysville, PA, USA) was performed in order to assess the presence of sleep disordered breathing. PSG examinations included a standard montage of electroencephalogram, electro‐oculogram, electromyogram (submental and bilateral tibial) and electrocardiogram signals. Respiratory events were recorded via combined oronasal thermistors (apneas) and nasal pressure transducer (hypopneas) and thoracic/abdominal strain gauges, while oxyhemoglobin saturation was assessed using a pulse oximeter placed on the index finger. Apnea was defined as a drop in peak airflow signal ≥90% of pre‐event baseline for at least 10 s, while hypopnea was defined as a drop in peak signal by ≥30% of pre‐event baseline for at least 10 s with oxyhemoglobin desaturation of at least 3% or an arousal recorded in the electroencephalogram.^19^ AHI was defined as the average number of apneas and hypopneas per hour of sleep recorded time. A diagnosis of OSA was considered at AHI ≥ 5 events/h of sleep plus OSA related symptoms or at AHI ≥ 15 events/h of sleep.^19^

#### 2.3.6 Treatment of OSA and COPD

Following the diagnosis of OSA, all patients were offered an in‐laboratory CPAP titration study with PSG to establish the appropriate pressure settings, which could eliminate all respiratory events and normalize patients' sleep architecture, as per AASM guidelines.^20^ CPAP devices with fixed pressure were provided to all patients who accepted treatment for their OSA. Good compliance to OSA treatment was defined as the average night use of CPAP for at least 4 h per night on at least 70% of the nights.^20^ Information regarding hours and nights of CPAP use and residual AHI was obtained following data download from the CPAP device. All patients received the optimal COPD treatment according to the GOLD report and the “ABCD” group classification.^8^ At study visits, all participants received detailed instructions regarding good compliance to CPAP and the proper daily use of inhalation devices. Finally, recommendations were made to all participants regarding smoking cessation, standard influenza and/or pneumococcal vaccination, as well as counselling for sleep hygiene, weight loss measures and daily physical activity (when appropriate).

### 2.4 Follow‐up measurements

Apart from the regular sleep monitoring of patients regarding their OSA management, a specific follow‐up protocol was performed at 12 months under treatment with CPAP. In particular, the following parameters were recorded before and at 12 months of CPAP treatment: anthropometric parameters, COPD symptoms (assessed by CAT and mMRC questionnaires), PFTs, arterial blood gases and 6MWT. A minimal clinically important difference for CAT was 2, while for 6MWT 54–80 m and for FEV_1_ 100 ml.^21^, ^22^, ^23^ Finally, moderate, and severe exacerbations and hospitalizations related to COPD during the course of the study were recorded based on patients' medical history and their medical records. Compliance to CPAP use was also recorded at 12 months with data retrieved from the patients' device.

### 2.5 Study outcomes

The primary outcome of the study was the effect of compliance to CPAP on the number of moderate and severe AECOPD at 12 months of treatment with CPAP compared to baseline. In addition, the effect of compliance to CPAP treatment on hospitalizations related to COPD exacerbations, PFTs, arterial blood gases, symptoms (assessed by CAT and mMRC questionnaires) and 6MWT was investigated at 12 months compared to baseline.

### 2.6 Statistical analysis

All data were analysed using the IBM Statistical Package for Social Sciences version 17.0 (SPSS Inc, Chicago, Il, 2008). Normality of distribution for continuous variables was tested by the Kolmogorov–Smirnov test. All variables were expressed as the median (25th–75th percentile). Comparisons were carried out with the paired student t‐test or the Wilcoxon signed‐rank test. Multivariate regression analysis, after adjustment for known risk factors of COPD exacerbation, was also applied to evaluate the association between CPAP compliance and COPD exacerbations. Statistical significance was defined by a two‐tailed *p* value of ≤0.05.

## 3 RESULTS

### 3.1 Baseline characteristics

**FIGURE 1:**
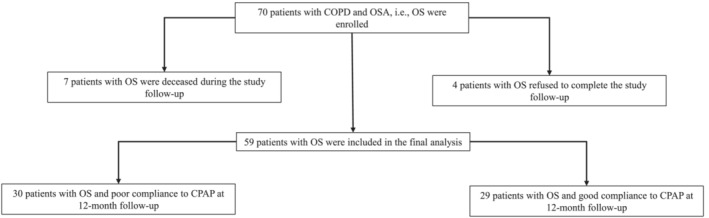
The study flowchart. COPD, chronic obstructive pulmonary disease; CPAP, continuous positive airway pressure; OS, overlap syndrome; OSA, obstructive sleep apnea

Α flow chart of the study is depicted in Figure 1. A total of 70 consecutive patients with COPD under optimal bronchodilation treatment, diagnosed with coexistent OSA between November 2017 and July 2020, were enrolled in the study. Of them, four patients refused to complete the study protocol, while seven (i.e., 10% of the total sample) died during the 12 months of follow‐up. According to patients' medical records, the deaths were not associated to COPD exacerbation or respiratory failure. Moreover, the group of patients who refused to complete the follow‐up did not differ in terms of anthropometrics, severity of COPD, and severity of OSA compared with the remaining patients.

The remaining 59 patients with OS (54 males) who received treatment with CPAP were followed up for 12 months for the study outcomes. Participants were treated with CPAP for 12 months and divided post hoc into two groups according to CPAP compliance: Group A included 29 patients with good CPAP compliance (5.5 [4.6–6.7] h/night) and group B included 30 patients with poor CPAP compliance (0.6 [0–3.3] h/night). All patients received optimal bronchodilator therapy based on their COPD GOLD stage and counselling for daily compliance to COPD treatment. Importantly, all patients had the same bronchodilation medication and found to be compliant to their medication during the study period.

At baseline, patients with good CPAP compliance had a higher AHI (*p* = 0.007) and FEV_1_ (=0.007) and performed better at the 6MWT (*p* = 0.022), compared to those with poor CPAP compliance. Moreover, patients with poor CPAP compliance had a significantly higher CAT score (*p* = 0.040) than those with good compliance. There were no differences between these two groups in terms of mMRC score and GOLD stage classification.

With regard to comorbidities, patients with poor CPAP compliance had a higher prevalence of cardiovascular disease (56.7% [*n* = 17] vs. 20.7% [*n* = 6], *p* = 0.05) and diabetes mellitus (43.3% [*n* = 13] vs. 13.8% [*n* = 4], *p* = 0.012) compared to patients with good CPAP compliance. The two groups did not differ in other comorbidities, namely, arterial hypertension, dyslipidemia, atrial fibrillation, chronic kidney disease, depression and gastroesophageal reflux (*p* > 0.05 for all). The comparison of baseline parameters between groups are displayed in Tables 1 and 2, while comparison of blood sample analysis is presented in Table S1.

**TABLE 1:** Comparison of anthropometric and sleep characteristics between patients with good and poor CPAP compliance at baseline

Characteristics	Patients with good CPAP compliance (*n* = 29)	Patients with poor CPAP compliance (*n* = 30)	*p*
Sex (male/female)	28/1	26/4	0.173
Age (years)	66 (54–70)	60.5 (56–67)	0.785
BMI (kg/m^2^)	36.8 (32.2–39.3)	40.4 (35.2–43.1)	0.053
Neck circumference (cm)	46 (44–50)	46.5 (43.8–50)	0.681
Waist circumference (cm)	127 (116.5–134)	132 (119–138)	0.391
Hip circumference (cm)	118 (109.5–122)	121 (111.8–129)	0.118
WHR	0.963 (0.893–1.019)	1.01 (0.893–1.071)	0.680
Smoking status			
Smoker	8 (27.6%)	12 (40%)	0.314
Former smoker	21 (72.4%)	18 (60%)	
TST (min)	320.5 (301.5–351)	308.3 (267.8–348.5)	0.439
N1 (% TST)	13.8 (3.3–22.2)	8.7 (4.9–17.7)	0.509
N2 (% TST)	69.7 (57.5–85.1)	73.4 (62.8–87)	0.219
N3 (% TST)	5.5 (0–11.5)	8.2 (1.2–13.4)	0.367
REM (% TST)	5.2 (0–12)	4.4 (0.8–10)	0.801
AHI (events/hour)	41.7 (30.9–53.6)	20.9 (17.9–42.9)	0.007*
Aver SpO_2_ (%)	90.9 (87.6–92)	91 (87.7–93.1)	0.946
Min SpO_2_ (%)	75 (63–77.5)	76 (68.8–82)	0.151
T < 90% (%)	23.9 (7–34.7)	24.7 (7.6–54.6)	0.671
Arousal index	6 (2–44.1)	7 (3–45)	0.836
Sleep efficiency (%)	86 (76.6–91)	79.8 (70.3–91.2)	0.314
ESS score	9 (6–15)	9 (6–16)	0.784

Abbreviations: AHI, apnea hypopnea index; Aver SpO_2_, average oxyhemoglobin saturation; BMI, body mass index; ESS, Epworth sleepiness scale; Min SpO_2_, minimum oxyhemoglobin saturation; N1, sleep stage 1; N2, sleep stage 2; N3, sleep stage 3; REM, rapid eye movement; TST, total sleep time; *T* < 90%, time with oxyhemoglobin saturation <90%; WHR, waist to hip ratio.

* Statistically significant *p* ≤ 0.05.

**TABLE 2:** Comparison of laboratory and pulmonary function testing between patients with good and poor CPAP compliance at baseline

Parameters	Patients with good CPAP compliance (*n* = 29)	Patients with poor CPAP compliance (*n* = 30)	*p*
Gas exchange			
pH	7.42 (7.41–7.45)	7.41 (7.40–7.43)	0.312
PaO_2_ (mmHg)	68 (64.5–79)	66.5 (60.8–76)	0.264
PaCO_2_ (mmHg)	44 (41.5–49)	46 (42–52)	0.475
HCO_3_^−^ (mmol/L)	28.5 (27.3–31.6)	29.1 (26.6–33.1)	0.820
Lung function			
FEV_1_ (% predicted)	73.6 (65.3–79.9)	61.5 (44.6–78.3)	0.007*
FVC (% predicted)	84 (76.1–93)	77.9 (59.5–91.2)	0.067
FEV_1_/FVC	69.1 (64.7–69.7)	67.8 (61.9–69.6)	0.158
RV (% predicted)	115.8 (97.4–137.6)	122 (101.6–150.5)	0.668
TLC (% predicted)	88.4 (81.9–96.1)	85 (76–102)	0.777
RV/TLC	126 (111–144.9)	136 (118.4–156.3)	0.107
DLCO (% predicted)	86.7 (68.3–96.1)	76.1 (60.3–88.1)	0.074
6MWT			
Distance (metres)	480 (385–540)	370 (240–495)	0.102
Distance (% predicted)	93 (85–107.5)	79 (65.5–95.3)	0.022*
SpO_2_ decrease (%)	2 (1–3)	2.5 (1–4)	0.239
CAT score	7 (5–11)	10 (8–11.5)	0.040*
mMRC score			
1 (*n*/%)	14 (48.3%)	10 (33.3%)	0.150
2 (*n*/%)	10 (34.5%)	10 (33.3%)	0.926
3 (*n*/%)	5 (17.2%)	9 (30%)	0.249
4 (*n*/%)	0 (0%)	1 (3.3%)	0.321
GOLD stage			
A (*n*/%)	12 (41.4%)	7 (23.3%)	0.138
B (*n*/%)	10 (34.5%)	13 (43.3%)	0.486
C (*n*/%)	0 (0%)	0 (0%)	‐
D (*n*/%)	7 (24.1%)	10 (33.3%)	0.436

Abbreviations: 6MWT, 6‐min walking test; CAT, COPD assessment test; DLCO, diffusing capacity for carbon monoxide; FEV_1_, forced expiratory volume in the first second; FVC, forced vital capacity; GOLD, global initiative for chronic obstructive lung disease; mMRC, modified medical research council; PaCO_2_, partial carbon dioxide pressure; PaO_2_, partial oxygen pressure; RV, residual volume; SpO_2_, oxyhemoglobin saturation; TLC, total lung capacity.

* Statistically significant *p* ≤ 0.05.

### 3.2 Follow‐up measurements

After 12 months of CPAP treatment residual AHI was 2.3 (1.3–5.8/h) for patients with good and 0 (0–7/) for patients with poor CPAP compliance. In addition, ESS scores reduced in both groups following treatment with CPAP (*p* < 0.001 for both). No changes in smoking status were observed in either group throughout the study period. The comparison of anthropometric characteristics before and after 12 months of CPAP therapy is shown in supplementary Table S2.

At 12 months, patients with good compliance to CPAP had improved pulmonary function and symptoms related to COPD as compared to baseline values. Specifically, increased partial oxygen pressure (*p* < 0.001) and decreased carbon dioxide partial pressure (*p* < 0.001) were noted, while FEV_1_ (*p* = 0.024), FVC (*p* = 0.008), total lung capacity (*p* = 0.024), diffusing capacity for carbon monoxide (*p* = 0.002) and RV/TLC (*p* = 0.003) were also improved. In the same group, patients covered a greater distance in the 6MWT (*p* < 0.001) and demonstrated a decrease in CAT score (*p* < 0.001).

In the group of patients with poor CPAP compliance, a decrease only in the RV/TLC ratio (*p* = 0.015) was observed after CPAP treatment compared to baseline. No other significant improvements were found in the poor compliant group. Comparison of pulmonary function parameters in both groups at baseline and after 12 months of CPAP treatment is shown in Table 3.

**TABLE 3:** Comparison of pulmonary function parameters between patients with good and poor CPAP compliance at baseline and after 12 months of treatment

Parameters	Patients with good CPAP compliance (*n* = 29)			Patients with poor CPAP compliance (*n* = 30)		
	Before	After	*p*	Before	After	*p*
Gas exchange						
pH	7.42 (7.41–7.45)	7.43 (7.41–7.44)	0.656	7.41 (7.40–7.43)	7.42 (7.41–7.44)	0.273
PaO_2_ (mmHg)	68 (64.5–79)	78 (72–83.5)	<0.001*	66.5 (60.8–76)	70.5 (61.8–76.3)	0.058
PaCO_2_ (mmHg)	44 (41.5–49)	41 (37–44.5)	<0.001*	46 (42–52)	44 (41–47)	0.094
HCO_3_^−^ (mmol/L)	28.5 (27.3–31.6)	27.2 (25.5–29.5)	0.003*	29.1 (26.6–33.1)	28.6 (27–31.1)	0.441
Lung function						
FEV_1_ (% predicted)	73.6 (65.3–79.9)	77 (69.6–86)	0.024*	61.5 (44.6–78.3)	64 (49.5–76.3)	0.131
FVC (% predicted)	84 (76.1–93)	90.9 (77.5–100.1)	0.008*	77.9 (59.5–91.2)	79.9 (62.6–92.5)	0.136
FEV_1_/FVC	69.1 (64.7–69.7)	69 (64–69.7)	0.313	67.8 (61.9–69.6)	66.8 (62–68.9)	0.842
RV (% predicted)	115.8 (97.4–137.6)	114 (98–126.8)	0.148	122 (101.6–150.5)	115.1 (98–148.9)	0.224
TLC (% predicted)	88.4 (81.9–96.1)	93.6 (85.2–99.9)	0.024*	85 (76–102)	84 (72.5–97.3)	0.137
RV/TLC	126 (111–144.9)	117 (107–127)	0.003*	136 (118.4–156.3)	125.7 (114.8–145.1)	0.015*
DLCO (% predicted)	86.7 (68.3–96.1)	87 (77–102)	0.002*	76.1 (60.3–88.1)	77.4 (70.5–90.3)	0.275
6MWT						
Distance (metres)	480 (385–540)	490 (402.5–550)	<0.001*	370 (240–495)	385 (267.5–490)	0.725
Distance (% predicted)	93 (85–107.5)	99 (90.5–116.5)	<0.001*	79 (65.5–95.3)	83 (68.3–95.8)	0.403
SpO_2_ decrease (%)	2 (1–3)	0 (0–1)	<0.001*	2.5 (1–4)	2.5 (2–4)	0.801
CAT score	7 (5–11)	3 (2–5)	<0.001*	10 (8–11.5)	10 (6–11)	0.381
mMRC score						
1 (*n*/%)	14 (48.3%)	25 (86.2%)	<0.001*	10 (33.3%)	8 (26.7%)	0.326
2 (*n*/%)	10 (34.5%)	3 (10.3%)	0.05*	10 (33.3%)	15 (50%)	0.096
3 (*n*/%)	5 (17.2%)	1 (3.5%)	0.043*	9 (30%)	6 (20%)	0.184
4 (*n*/%)	0 (0%)	0 (0%)	‐	1 (3.3%)	1 (3.3%)	1

Abbreviations: 6MWT, 6‐min walking test; CAT, COPD assessment test; DLCO, diffusing capacity for carbon monoxide; GOLD, global initiative for chronic obstructive lung disease; FEV_1_, forced expiratory volume in the first second; FVC, forced vital capacity; mMRC, modified medical research council; PaCO_2_, partial carbon dioxide pressure; PaO_2_, partial oxygen pressure; RV, residual volume; SpO_2_, oxyhemoglobin saturation; TLC, total lung capacity.

* Statistically significant *p* ≤ 0.05.

A total of 32 exacerbations were observed during the 12 months prior to therapy, and that was decreased to a total of 20 exacerbations after 12 months of CPAP treatment. Similarly, a total of 18 hospitalizations due to COPD exacerbations were observed during the 12 months prior to treatment and that was decreased to 5 hospitalizations after 12 months of CPAP treatment. More specifically, in patients with good compliance to CPAP there was a decrease in COPD exacerbations from baseline to 12 months (5 after vs. 17 before treatment, *p* = 0.001), but not in those with poor compliance to CPAP (15 after vs. 15 before treatment *p* = 1). Similarly, hospitalizations were decreased after treatment with CPAP in patients with good CPAP compliance from baseline to 12 months (0 after vs. 6 before treatment, *p* = 0.012), while there was a non‐significant decrease in those with poor compliance (5 after vs. 12 before treatment, *p* = 0.056).

A negative association between hours of CPAP use (*r* = −0.259, *p* = 0.047) and COPD exacerbations was noted. Multivariate regression analysis, after adjustment for known anthropometric (age, BMI, exacerbations in the year prior to CPAP treatment and smoking status), pulmonary function (FEV_1_, PaO_2_ and CAT score) and polysomnographic parameters (AHI, average oxyhemoglobin saturation during sleep and time with oxyhemoglobin saturation <90%) showed that patients with poor CPAP compliance had 0.362 COPD exacerbations more than those with good compliance (β = 0.362, 95% CI: 0.075–0.649, *p* = 0.015).

## 4 DISCUSSION

The present study demonstrates the beneficial effects of CPAP compliance οn several outcomes in patients with OS. This study reports that exacerbations and hospitalizations in COPD patients with newly diagnosed OSA are reduced following 12 months of treatment with CPAP. In particular, patients with poor CPAP compliance have a higher risk of COPD exacerbations. Additionally, indices of pulmonary function, including parameters of static volumes and blood gas exchange parameters are improved in the CPAP compliant group compared to the non‐compliant group. Likewise, symptoms of COPD, as assessed by the CAT and mMRC questionnaires, are ameliorated when OSA is effectively treated.

Acute exacerbations of COPD are a significant feature of the natural history of COPD, resulting in faster disease progression and pulmonary function decline, while they cause a negative impact on healthcare systems contributing to a huge financial burden.^7^ Existing evidence highlights the burden of OS on AECOPD and underline the significant role of PAP use on COPD outcomes.^11^ Importantly, patients with OS are exposed at increased risk of exacerbations and hospitalizations related to COPD.^4^ PAP treatment is of utmost importance during AECOPD, as it reduces patients' mortality,^24^ and has proven useful for stable chronic hypercapnic patients reducing the risks of readmission or death.^25^ In addition, CPAP stands as the treatment of choice in patients with OS as well.^4^, ^8^

In the present study, treatment with CPAP reduced the number of AECOPD in a group of patients with OS. Particularly, it was noted that the number of COPD exacerbations and hospitalizations significantly decreased in OS patients following 12 months of treatment with good compliance to CPAP. Previous studies reported that CPAP reduced COPD exacerbations in patients with OS and had some role on patients' survival, compared to subjects with COPD and untreated OSA.^4^, ^26^ Marin et al.^4^ demonstrated that over a median follow‐up of 9.4 years OS patients without CPAP had a higher risk for first‐time hospitalization due to COPD exacerbations, compared with COPD patients (RR: 1.70; 95% CI: 1.21–2.38) and increased mortality risk in respect with the COPD group (RR: 1.70; 95% CI: 1.21–2.38).^4^ A retrospective study involving 225 with OS, who were followed up for 42 months, reported that OS patients with at least one COPD exacerbation had fewer time spent with CPAP than matched OS patients without history of exacerbation (*p* = 0.001).^26^ In line with these findings, our results show that sufficient compliance with CPAP could exert beneficial effects on the number of AECOPD and hospitalizations in patients with OS.

In our study, AECOPD correlated with poor compliance to CPAP treatment and hours of CPAP use. Several factors predict the risk of AECOPD like history of exacerbations in the previous year, higher CAT scores and lower Interleukin‐15 (IL‐15) and elevated Interleukin‐8 (IL‐8) levels.^27^, ^28^ In the context of OS, previous studies have investigated factors related to AECOPD and mortality; among others, treatment of the OSA with CPAP is a key element. Jaoude and El‐Solh^26^ revealed that poor compliance to CPAP was an independent predictor of at least one AECOPD and all‐cause mortality.^26^ Stanchina et al.^29^ examined 227 patients with OS and reported that longer time spent with CPAP reduced mortality, especially when CPAP is used for 6 to 8 h in comparison to 0 to 2 h per night. Another study showed that compliance to CPAP had beneficial effect on survival in hypercapnic patients with OS but not in those with normocapnia.^30^ Collectively, our findings as well as results from previous studies indicate that good adherence to CPAP plays a protective role against AECOPD and mortality independently of other cofounders.

In our study, patients with good compliance to CPAP treatment had improved pulmonary function testing, as assessed by the FEV_1_ and the static lung volumes. This group of patients performed better in 6MWT and presented reduced scores in CAT and mMRC questionnaires. Furthermore, the group of patients with poor compliance to CPAP demonstrated an improvement only in RV/TLC. As already mentioned, all participants received the optimal bronchodilation treatment, which did not change during the follow‐up. This fact denotes that CPAP on top of the bronchodilation treatment may have also improved patients' lung function and exercise capacity. Nevertheless, it should be noted that the minimal clinically important difference was achieved only for CAT score and neither for 6MWT nor for FEV_1_ in the compliant group; a larger number of participants could have also reached these targets. These findings are in line with the existing body of literature. Indeed, CPAP improves airflow limitation and dynamic hyperinflation, gas exchange and walking capacity.^31^, ^32^ In the study of Lopes et al.,^33^ CPAP improved respiratory mechanics and the work of breathing by reducing lung volumes and airway resistance and minimizing hyperinflation in COPD patients. An earlier study addressing the effects of CPAP in patients with OS showed that partial pressures of oxygen and dioxide levels as well as FEV_1_ and FVC were improved after 6 months of CPAP therapy, findings which were more profound in the hypercapnic group.^31^ In the study of Wang et al.,^32^ short‐term treatment with CPAP resulted in increased walking capacity and reduced dyspnea, as documented by the Borg scale, compared with baseline measurements in patients with OS.

The present study is subject to several limitations. First, this is a single centre observational study, including a relatively small number of participants and hence robust evidence cannot be extracted regarding the definite role of CPAP on the COPD outcomes. Second, there was an overrepresentation of males over females, but this may reflect the epidemiological data on OS which confirms higher prevalence and relative burden of both diseases (i.e., COPD and OSA) among males. Nonetheless, there was an equal distribution of patients in both groups (compliant vs. non‐compliant) and there were no differences between groups in several characteristics. Moreover, the group of patients with good compliance to CPAP had a higher AHI than the group with poor compliance and this might have also resulted in better compliance to CPAP therapy in this group of patients. However, the two groups did not differ at baseline in other sleep parameters such as the ESS and the nocturnal oxygen indices. In addition, there were no patients with mild OSA available to participate in the present study. This fact emerges as an opportunity for further research in future studies. Finally, included patients with COPD also classified in all GOLD stages, except for C group. The latter indicates the epidemiological data of the relatively small number of patients pertaining to this group.

The findings of the study are strengthened by the fact that it includes a well characterized patient cohort, which is treated according to the latest COPD and OSA guidelines. Moreover, the present study underlines the benefits of good compliance to CPAP in several COPD outcomes on top of the standard bronchodilation therapy. The design of the study anticipated the inclusion of consecutive patients with COPD at stable condition as they referred for evaluation of sleep disordered breathing in daily clinical practice.

In summary, the results of this real‐life observational study denote that CPAP use reduces the number of AECOPD and is beneficial for several COPD related outcomes in a cohort of OS patients. Moreover, this study addresses the central role of compliance to OSA treatment with CPAP on the management of these patients, a finding that merits further research in the field of OS.

## CONFLICT OF INTEREST

The authors do not have any conflict of interest to disclose.

## ETHICS STATEMENT

This study was approved by the ethics committee of University General Hospital of Alexandroupolis, Greece (IRB:14‐1‐27.01.2017). Written informed consent was obtained from all participants upon enrollment to the study.

## AUTHOR CONTRIBUTIONS

AV performed data collection and data verification, drafted the manuscript and compiled edits. KA performed data collection and contributed to the initial draft of the manuscript. PS contributed to both the design of the analysis and to revisions of the manuscript. SA, MF, SS and MEF contributed to revisions of the manuscript. AV and PS conceived the idea and provided edits to the manuscript. All authors read and approved the final manuscript.

## Supporting information


**Table S1.** Comparison of laboratory parameters between patients with good and poor CPAP compliance at baseline.
**Table S2.** Comparison of anthropometric parameters between patients with good and poor CPAP compliance at baseline and after 12 months of treatment with CPAP.

## Data Availability

The data that support the findings of this study are available from the corresponding author upon reasonable request.
