# Supplementary material for: Effect of compliance to continuous positive airway pressure on exacerbations, lung function and symptoms in patients with chronic obstructive pulmonary disease and obstructive sleep apnea (overlap syndrome)
Source: Clin Respir J. 2023 Jan 12;17(3):165–75. doi: 10.1111/crj.13580 (PMC9978906; doi:10.1111/crj.13580)
Supplement: Supplementary file 1 — Table S1. Comparison of laboratory parameters between patients with good and poor CPAP compliance at baseline. Table S2. Comparison of anthropometric parameters between patients with good and poor CPAP compliance at baseline and after 12 months of treatment with CPAP. [file CRJ-17-165-s001.docx]

**Supplementary Table 1.** Comparison of laboratory parameters between patients with good and poor CPAP compliance at baseline.

| **Parameters** | **Patients with good CPAP compliance**  **(n = 29)** | **Patients with poor CPAP compliance**  **(n = 30)** | **p** |
| --- | --- | --- | --- |
| **WBC (x10^3^/μL)** | 7.210 (6.425 – 8.510) | 8.055 (6.588 – 9.655) | 0.114 |
| **Creatinine (mg/dL)** | 0.90 (0.85 – 1.1) | 0.95 (0.88 – 1.1) | 0.711 |
| **Fibrinogen (mg/dL)** | 353 (290 – 387) | 332.5 (270 – 382.5) | 0.611 |
| **CRP (mg/dL)** | 0.35 (0.16 – 0.7) | 0.5 (0.19 – 1.16) | 0.189 |
| **Glucose (mg/dL)** | 104 (94 – 128.5) | 111 (97.5 – 161) | 0.292 |
| **Triglycerides (mg/dL)** | 126 (108 – 205.5) | 156.5 (128.8 – 223.3) | 0.117 |
| **Cholesterol (mg/dL)** | 184 (171 – 217.5) | 192 (151.8 – 219) | 0.974 |
| **HDL (mg/dL)** | 48 (41.5 - 55.5) | 47 (40.8 – 55) | 0.867 |
| **LDL (mg/dL)** | 104.6 (81.9 – 138.3) | 97.3 (78.7 – 139.1) | 0.573 |

**Abbreviations**: CPAP: continuous positive airway pressure; CRP: C-reactive protein; HDL: high density lipoprotein; LDL; low density lipoprotein; WBC: white blood cells.

**Supplementary Table 2.** Comparison of anthropometric parameters between patients with good and poor CPAP compliance at baseline and after 12 months of treatment

|  | **Patients with good CPAP compliance**  **(n = 29)** | | | **Patients with poor CPAP compliance**  **(n = 30)** | | |
| --- | --- | --- | --- | --- | --- | --- |
| **Characteristics** | **Before** | **After** | **p** | **Before** | **After** | **p** |
| **BMI (kg/m2)** | 36.8 (32.2 – 39.3) | 35.5 (31.2 – 39.2) | **0.024** | 40.4 (35.2 – 43.1) | 39.2 (34.2 – 42.7) | 0.338 |
| **Neck circumference (cm)** | 46 (44 – 50) | 47 (43 – 49) | 0.100 | 46.5 (43.8 – 50) | 45 (42 – 49) | 0.329 |
| **Waist circumference (cm)** | 127 (116.5 – 134) | 126 (115 – 133) | 0.290 | 132 (119 – 138) | 128 (118.8 – 139.8) | 0.312 |
| **Hip circumference (cm)** | 118 (109.5 – 122) | 115 (107.5 – 124.5) | 0.398 | 121 (111.8 – 129) | 123 (112.5 – 137.3) | 0.096 |
| **WHR** | 0.963 (0.893 – 1.019) | 1.040 (1.009 – 1065) | **<0.001** | 1.01 (0.893 – 1.071) | 1.025 (0.988 – 1.078) | **0.017** |
| **ESS** | 9 (6-15) | 2 (1-4.5) | **<0.001** | 9 (6-16) | 4.5 (2-8.3) | **<0.001** |

**Abbreviations:** BMI: Body mass index; CPAP: continuous positive airway pressure; ESS: Epworth severity scale; WHR: Waist to hip ratio.
